# More than just a pool

**DOI:** 10.7554/eLife.61397

**Published:** 2020-09-11

**Authors:** Amanda Cinquin, Olivier Cinquin

**Affiliations:** Department of Developmental and Cell Biology, Center for Complex Biological Systems, University of California, IrvineIrvineUnited States

**Keywords:** stem cell niche, asymmetric cell division, gonadal sheath, distal tip cell, germ cell, niche exit, *C. elegans*

## Abstract

An intricate stem cell niche boundary formed by finger-like extensions generates asymmetry in stem cell divisions.

**Related research article** Gordon KL, Zussman JW, Li X, Miller C, Sherwood DR. 2020. Stem cell niche exit in *C. elegans* via orientation and segregation of daughter cells by a cryptic cell outside the niche. *eLife* **9**:e56383. doi: 10.7554/eLife.56383

Stem cells have the ability to divide and self-renew or specialize into many different types of cells that replenish tissues and organs. Historically, and based largely on blood stem cells, divisions have been thought to be asymmetric, resulting in two daughter cells with different fates: an identical, slow-cycling stem cell and a faster-cycling progenitor cell committed to differentiation. However, self-renewal of many tissues, such as the intestine, is ensured by cells that do not display strong division asymmetry and are instead organized as pools of progenitor cells. Daughter cells of these progenitors frequently do not appear to differ in their likelihood to self-renew or specialize (Post and Clevers, 2019).

Establishing the design principles underlying such progenitor pools is key to understanding how continuous self-renewal is maintained. Now, in eLife, Kacy Gordon and colleagues from the University of North Carolina and Duke University report new insights about stem cell division in the nematode *Caenorhabditis elegans* (Gordon et al., 2020).

In *C. elegans*, germ stem cells – which ensure the production of oocytes and sperm – reside at one end of tube-shaped gonads in what is known as the progenitor zone. The progenitor zone is capped by a large cell called the distal tip cell. The distal tip cell controls the proliferation of germ stem cells, and its finger-like extensions are thought to communicate with these cells (Fitzgerald and Greenwald, 1995; Byrd et al., 2014). Proximal sheath cells (Sh1 cells) surround the gonads and wrap the differentiating germ stem cells exiting the progenitor zone.

Germ stem cells within the progenitor zone show some variation in specialization (the cells closest to the proximal end of the gonads start expressing genes associated with the differentiation of reproductive cells). But the orientation of progenitor division was reported to be largely random, compatible with the idea that the progenitor zone, or at least a distal portion thereof, forms a ‘bag’ of mostly equivalent proliferating cells – with the most proximal being randomly pushed out and differentiating. The speed of the cell cycle is largely similar among progenitors, apparently furthering the notion that the differentiation process is not controlled by division asymmetry (Maciejowski et al., 2006; Crittenden et al., 2006; Jaramillo-Lambert et al., 2007; Chiang et al., 2015; Rosu and Cohen-Fix, 2017).

To investigate how the cell fate of germ stem cells is regulated, Gordon et al. used fluorescent labeling of both the distal tip cell and the Sh1 cells and tracked the dividing germ stem cells. This revealed that both the distal tip cell and Sh1 cells intercalate long protrusions that contact the germ stem cells (Figure 1a). Unexpectedly, most cell divisions happened at the distal tip cell-Sh1 interface. Most strikingly, these divisions were often asymmetrical, with one daughter cell staying in contact with the distal tip cell and the other one with Sh1 cells – turning the idea on its head that the progenitor zone is a pool of randomly proliferating cells. Manipulation of the cytoskeleton-related gene expression further suggested that a tightly knit interface between the distal tip cell and Sh1 cells may be necessary for robust proliferation. However, this does not rule out that this interface could also respond to signals from dividing germ stem cells. This interface may also play a role in positioning gene expression patterns within the progenitor zone.

**Figure 1.**
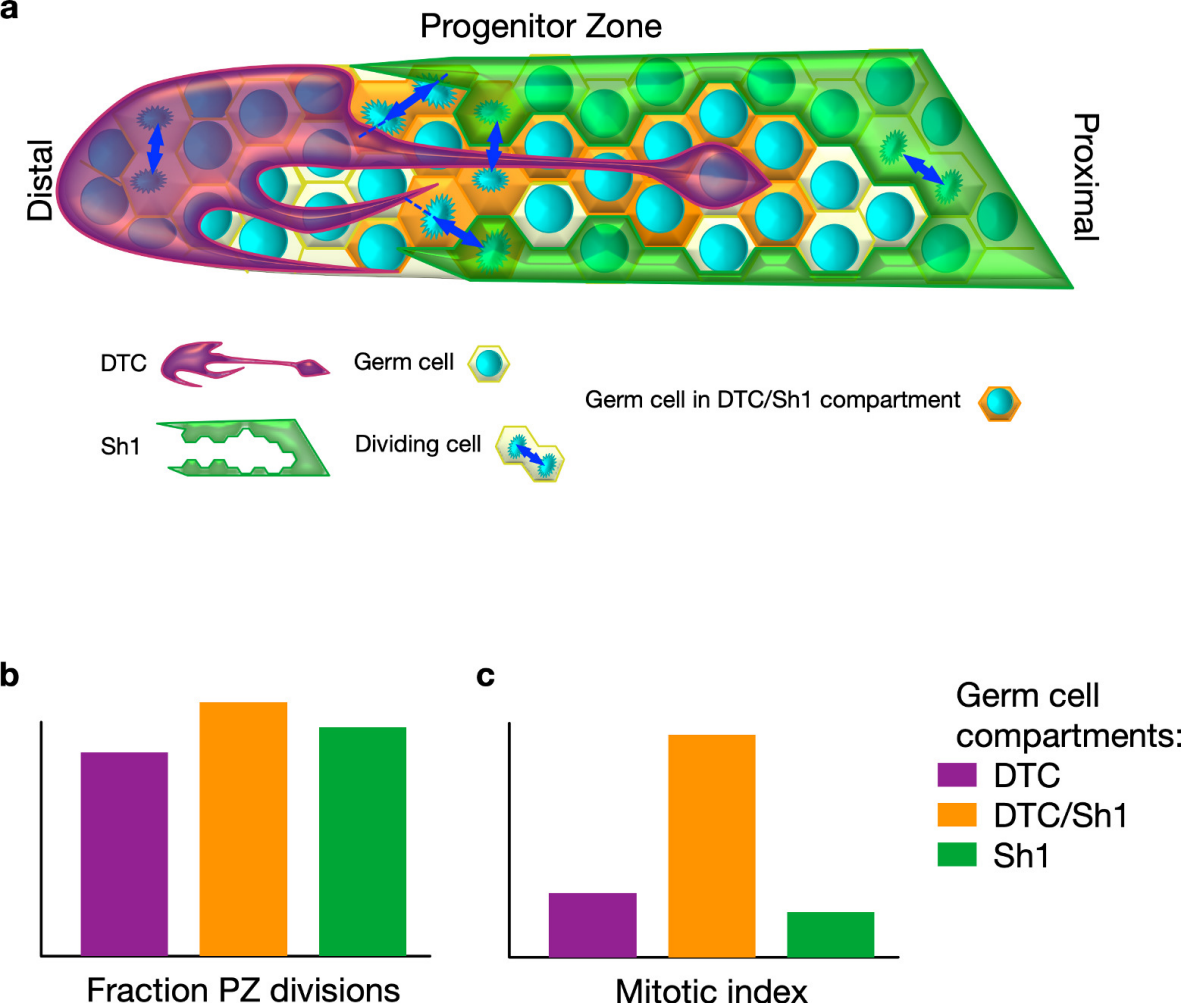
Stem cell division in *Caenorhabditis elegans*. (**A**) In *C. elegans*, germ stem cells reside in a niche formed by the distal tip cell (DTC) and are wrapped by the proximal sheath cells (Sh1) as they move proximally and differentiate. The DTC forms protrusions that may communicate with germ stem cells. Gordon et al. discovered that Sh1 cells also have finger-like extensions that intercalate with the DTC and contact progenitors. (**B**) Cell division takes place in three different compartments within the progenitor zone (PZ): one covered by the DTC (purple) , one at the interface of the DTC and Sh1 (orange), and one covered by Sh1 (green). Divisions are often asymmetric in that each daughter maintains contact with one of DTC or Sh1. (**C**) Germ cells contacting both the DTC and Sh1 cells contribute a substantial fraction of overall progenitor generation because they have a higher proportion of dividing cells to non-dividing cells compared to the other compartments.

The work of Gordon et al. illustrates that a niche is more than just a region that accommodates a given number of stem cells or that serves as a punctual source of a self-renewal signals (Schofield, 1978). Rather, these experiments have unearthed hidden layers of control and thus provide a stepping stone to future research unraveling unknown mechanisms underlying cell fate determination. For example, what is the purpose of asymmetric cell division in this specific area? Could the intricate shape of the niche enlarge the surface area and so increase the number of asymmetric divisions in this progenitor zone? This asymmetry, even if it does not anchor stem cells, could still shape clonal dynamics in a way that helps minimize mutations and prevent premature senescence of germline stem cells (Cairns, 2006; Chiang et al., 2015; Cinquin et al., 2016).

In the future, it will be important to study germ stem cells below the gonad surface, which may have different behaviors;, and to assay the impact of asymmetric division on the dynamics of stem cell clones. It remains to be seen if protrusions similar to those of the distal tip cell and those of other cell types such as embryonic stem cells (Ramírez-Weber and Kornberg, 1999; Inaba et al., 2015; Junyent et al., 2020), are a prevalent feature of stem cell niches. Such structures could have remained hidden because of imaging difficulties, and may represent a hub for asymmetric cell divisions in tissues currently viewed as lacking those features.
